# Supplementary material for: Transcription Terminator-Mediated Enhancement in Transgene Expression in Maize: Preponderance of the AUGAAU Motif Overlapping With Poly(A) Signals
Source: Front Plant Sci. 2020 Oct 14;11:570778. doi: 10.3389/fpls.2020.570778 (PMC7591816; doi:10.3389/fpls.2020.570778)
Supplement: Supplementary Figure 1 — Protein sequence alignment of Z. mays (ZM) Ubi1, S. italica Ubi2, B. distachyon Ubi1 and B. distacyon Ubi1-C UBQ genes. The highlighted amino acids represent consensus among two (turquoise) or all four (yellow) sequences. [file DataSheet_1.pdf]

Supplementary Figure 1

```
                                                                                                    Section 1
                      (1) 1        10        20        30        40        50        60        70              88
            ZM Ubi1   (1) MQIFVKTLTGKTITLEVESSDTIDNVKAKIQDKEGIPPDQQRLIFAGKQLEDGRTLADYNIQKESTLHLVLRLRGGMQIFVKTLTGKT
     S italica Ubi2   (1) MQIFVKTLTGKTITLEVESSDTIDNVKAKIQDKEGIPPDQQRLIFAGKQLEDGRTLADYNIQKESTLHLVLRLRGGMQIFVKTLTGKT
  B distachyon Ubi1   (1) ----------------------------------------------------------------------------------------
 B distachyon Ubi1C   (1) ----------------------------------------------------------------------------------------
          Consensus   (1) MQIFVKTLTGKTITLEVESSDTIDNVKAKIQDKEGIPPDQQRLIFAGKQLEDGRTLADYNIQKESTLHLVLRLRGGMQIFVKTLTGKT
                                                                                                    Section 2
                     (89) 89         100       110       120       130       140       150       160            176
            ZM Ubi1  (89) ITLEVESSDTIDNVKAKIQDKEGIPPDQQRLIFAGKQLEDGRTLADYNIQKESTLHLVLRLRGGMQIFVKTLTGKTITLEVESSDTID
     S italica Ubi2  (89) ITLEVESSDTIDNVKAKIQDKEGIPPDQQRLIFAGKQLEDGRTLADYNIQKESTLHLVLRLRGGMQIFVKTLTGKTITLEVESSDTID
  B distachyon Ubi1   (1) ----------------------------------------------------------------MQIFVKTLTGKTITLEVESSDTID
 B distachyon Ubi1C   (1) ----------------------------------------------------------------MQIFVKTLTGKTITLEVESSDTID
          Consensus  (89) ITLEVESSDTIDNVKAKIQDKEGIPPDQQRLIFAGKQLEDGRTLADYNIQKESTLHLVLRLRGGMQIFVKTLTGKTITLEVESSDTID
                                                                                                    Section 3
                    (177) 177          190       200       210       220       230       240       250         264
            ZM Ubi1 (177) NVKAKIQDKEGIPPDQQRLIFAGKQLEDGRTLADYNIQKESTLHLVLRLRGGMQIFVKTLTGKTITLEVESSDTIDNVKAKIQDKEGI
     S italica Ubi2 (177) NVKAKIQDKEGIPPDQQRLIFAGKQLEDGRTLADYNIQKESTLHLVLRLRGGMQIFVKTLTGKTITLEVESSDTIDNVKAKIQDKEGI
  B distachyon Ubi1  (25) NVKAKIQDKEGIPPDQQRLIFAGKQLEDGRTLADYNIQKESTLHLVLRLRGGMQIFVKTLTGKTITLEVESSDTIDNVKAKIQDKEGI
 B distachyon Ubi1C  (25) NVKAKIQDKEGIPPDQQRLIFAGKQLEDGRTLADYNIQKESTLHLVLRLRGGMQIFVKTLTGKTITLEVESSDTIDNVKAKIQDKEGI
          Consensus (177) NVKAKIQDKEGIPPDQQRLIFAGKQLEDGRTLADYNIQKESTLHLVLRLRGGMQIFVKTLTGKTITLEVESSDTIDNVKAKIQDKEGI
                                                                                                    Section 4
                    (265) 265  270        280       290       300       310       320       330       340         352
            ZM Ubi1 (265) PPDQQRLIFAGKQLEDGRTLADYNIQKESTLHLVLRLRGGMQIFVKTLTGKTITLEVESSDTIDNVKAKIQDKEGIPPDQQRLIFAGK
     S italica Ubi2 (265) PPDQQRLIFAGKQLEDGRTLADYNIQKESTLHLVLRLRGGMQIFVKTLTGKTITLEVESSDTIDNVKAKIQDKEGIPPDQQRLIFAGK
  B distachyon Ubi1 (113) PPDQQRLIFAGKQLEDGRTLADYNIQKESTLHLVLRLRGGMQIFVKTLTGKTITLEVESSDTIDNVKAKIQDKEGIPPDQQRLIFAGK
 B distachyon Ubi1C (113) PPDQQRLIFAGKQLEDGRTLADYNIQKESTLHLVLRLRGGMQIFVKTLTGKTITLEVESSDTIDNVKAKIQDKEGIPPDQQRLIFAGK
          Consensus (265) PPDQQRLIFAGKQLEDGRTLADYNIQKESTLHLVLRLRGGMQIFVKTLTGKTITLEVESSDTIDNVKAKIQDKEGIPPDQQRLIFAGK
                                                                                                    Section 5
                    (353) 353    360       370       380       390       400       410       420       430       440
            ZM Ubi1 (353) QLEDGRTLADYNIQKESTLHLVLRLRGGMQIFVKTLTGKTITLEVESSDTIDNVKAKIQDKEGIPPDQQRLIFAGKQLEDGRTLADYN
     S italica Ubi2 (353) QLEDGRTLADYNIQKESTLHLVLRLRGGMQIFVKTLTGKTITLEVESSDTIDNVKAKIQDKEGIPPDQQRLIFAGKQLEDGRTLADYN
  B distachyon Ubi1 (201) QLEDGRTLADYNIQKESTLHLVLRLRGGMQIFVKTLTGKTITLEVESSDTIDNVKAKIQDKEGIPPDQQRLIFAGKQLEDGRTLADYN
 B distachyon Ubi1C (201) QLEDGRTLADYNIQKESTLHLVLRLRGGMQIFVKTLTGKTITLEVESSDTIDNVKAKIQDKEGIPPDQQRLIFAGKQLEDGRTLADYN
          Consensus (353) QLEDGRTLADYNIQKESTLHLVLRLRGGMQIFVKTLTGKTITLEVESSDTIDNVKAKIQDKEGIPPDQQRLIFAGKQLEDGRTLADYN

                                                                                                    Section 6
                    (441) 441      450       460       470       480       490       500       510               528
            ZM Ubi1 (441) IQKESTLHLVLRLRGGMQIFVKTLTGKTITLEVESSDTIDNVKAKIQDKEGIPPDQQRLIFAGKQLEDGRTLADYNIQKESTLHLVLR
     S italica Ubi2 (441) IQKESTLHLVLRLRGGMQIFVKTLTGKTITLEVESSDTIDNVKAKIQDKEGIPPDQQRLIFAGKQLEDGRTLADYNIQKESTLHLVLR
  B distachyon Ubi1 (289) IQKESTLHLVLRLRGGMQIFVKTLTGKTITLEVESSDTIDNVKAKIQDKEGIPPDQQRLIFAGKQLEDGRTLADYNIQKESTLHLVLR
 B distachyon Ubi1C (289) IQKESTLHLVLRLRGGMQIFVKTLTGKTITLEVESSDTIDNVKAKIQDKEGIPPDQQRLIFAGKQLEDGRTLADYNIQKESTLHLVLR
          Consensus (441) IQKESTLHLVLRLRGGMQIFVKTLTGKTITLEVESSDTIDNVKAKIQDKEGIPPDQQRLIFAGKQLEDGRTLADYNIQKESTLHLVLR
                                                                                                    Section 7
                    (529) 529  534
            ZM Ubi1 (529) LRGGQ-
     S italica Ubi2 (529) LRGGQ-
  B distachyon Ubi1 (377) LRGGQ-
 B distachyon Ubi1C (377) LRGGQ-
          Consensus (529) LRGGQ
```
