# Supplementary material for: Transcription Terminator-Mediated Enhancement in Transgene Expression in Maize: Preponderance of the AUGAAU Motif Overlapping With Poly(A) Signals
Source: Front Plant Sci. 2020 Oct 14;11:570778. doi: 10.3389/fpls.2020.570778 (PMC7591816; doi:10.3389/fpls.2020.570778)
Supplement: Supplementary Figure 2 — Nucleotide sequences of the BdUbi1-C (2A), BdUbi1 (2B) and SiUbi2 (2C) genes depicting putative promoters (underlined sequences distal of ATG contain TSS and upstream sequences), 5′ UTR (upper case), intron (grey highlighted), protein coding sequences (italicized), and TT regions (downstream of TAA, a translational stop codon). [file DataSheet_2.pdf]

Supplementary Figure 2

2A

ctgctcgttcagcccacagtaacacgccgtgcgacatgcagatgccctccaccacgccgaccaaccccaagtccgccgcgctcgtccacggcgccatccgcatccgcg
cgtcaacgtcatccggaggaggcgagcgcgatgtcgacggccacggcggcggacacgacggcgacgccccgactccgcgcgcgcgtcaaggctgcagtggcg
tcgtggtggccgtccgcctgcacgagatccccgcgtggacgagcgccgcctccacccagcccctatatcgagaaatcaacggtgggctcgagctcctcagcaacctcc
ccacccccccttccgaccacgctcccttcccccgtgcccctcttctccgtaaacccgagccgccgagaacaacaccaacgaaagggcgaagagaatcgccatagaga
ggagatgggcggaggcggatagtttcagccattcacggagaaatggggaggagagaacacgacatcatacggacgcgaccctctagctggctggctgtcctaaaga
atcgaacggaatcgctgcgccaggagaaaacgaacggtcctgaagcatgtgcgcccggttcttccaaaacacttatctttaagattgaagtagtatatatgactgaaa
tttttacaaggtttttccccataaaacaggtgagcttatctcatccttttgtttaggatgtacgtattatatatgactgaatattttttattttcattgaatgaagattttcgac
cccccaaaaataaaaaacggagggagtacctttgtgccgtgtatatggactagagccatcgggacgtttccggagactgcgtggtgggggcgatggacgcacaacg
accgcattttcggttgccgactcgccgttcgcatctggtaggcacgactcgtcgggttcggctcttgcgtgagccgtgacgtaacagacccgttctcttcccccgtctggc
catccataaatccccccctccatcggcttccctttcctcaatccagcaccctgattCCGATCGAAAAGTCCCCGCAAGAGCAAGCGACCGATCTCGTG
AATCTCCGTCAAGgtatgcagcctcgcttcctcctcgctaccgtttcaattctggagtaggtcgtagaggataccatgttgatttgacagagggagtagattagat
acttgtagatcgaagtgcggatgttccatggtagatgataccatgttgatttcgattagatcggattaaatctttgtagatcgaagtgcgcatgttccatgaattgcctgt
taccagtagattcaagtttttctgtgttatagaggtgggatctactcgttgagatgattagctcctagaggacaccatgccgttttggaaaatagatcagaaccgtgtag
atcgatgtgagcatgtgttcctgtagatccaagttctttcgcatgttactagttgtgatctattgtttgtgtaatacgctctcgatctatccgtgtagatttcactcgattact
gttactgtggcttgatcgttcatagttgttcgttaggtttgatcgaacagtgtctgaacctaattggatatgtattcttgatctatcaacgtgtaggtttcagtcatgtattta
tgtactccctccgtcccaaattaactgacgtggattttgtataagaatctatacaaatccatgtcagttaattcgggatggagtaccatattcaataatttgtttattgctg
tccacttatgtaccatatgtttgttgttcctcatgtggattctactaattatcattgattggtgatcttctattttgctagtttcctagctcaatctggttattcatgtagatgtg
ttgttgaaatcggagaccatgcttgttattagatagtttattgcttatcagtttcatgttctggttgatgcaacacatattcatgttcgctatctggttgctgcttgatattctc
tgatttacattcattataagaatatattctgctctggttgttgcttctcatgactttacctactcggtaggtgacttaccttttggtttacaattgtcaactatgcag***ATG****ca*
gatctttgtgaagaccctcaccggcaagaccatcacccttgaggtcgagtcttctgatacgatcgacaatgtcaaggcaaagatccaggacaaggagggcatc
cccccggaccagcagcgtctcatcttcgccgggaagcagctggaggatggccgcaccctggcagattacaacatccagaaggagtccaccctccatctggtgct
caggctcaggggtggcatgcaaatctttgtgaagacccttactggcaagaccatcacactcgaggtcgagtcgtctgacacgatcgacaatgtgaaggcaaa
gatccaggacaaggagggcatcccccagaccagcagcgcctcatctttgctggcaagcaactggaagacggtcgcaccctggcagattacaatatccagaa
ggagtccaccttgcaccttgtgcttcgcctccgtggtggcatgcaaatctttgtcaagaccttgacaggcaagaccattactctggaggtcgagtcgtctgacaca
atcgataatgtgaaggcgaagatccaggacaaggagggaattccaccggaccagcagcgcctaatctttgctggcaaacagcttgaggatggccgcaccct
ggcagattacaacatccagaaagaatccactctgcacctggtgcttcgcctccgtggtggcatgcagatctttgtcaagaccttgacagggaagacaatcacac
tggaggtcgagtcgtctgacacaatcgataatgtgaaggcgaagatccaggacaaggagggcattccaccggaccagcagcgccttatcttcgccggcaagc
agcttgaggatggccgcacccttgctgattacaatatccagaaggaatccaccctgcacctggtgcttcgcctccgtggtggcatgcagatctttgtcaagacttt
gaccgggaagaccattacactggaggttgaatcttcagacaccatcgacaacgtgaaggcgaagatccaggacaaggagggcatcccccagaccagcag
cgcctgatctttgctggtaagcagcttgaggatggacgcactctggcggattataacatccagaaggagtctaccctacacctggtgctccgcctccgtggtggc
*cag****TAA***gtttgtcaaaaactggcctacagtctgctgcccctgttggtctgccccttggaagtagtcgtgtctatggttatgtgagaagtcgttgtgttctttctaatcccg
tactgtttgtgtgaacatctgctgctgtcgtattgcatcgtgaagaatcctgttatgaataagtgaacatgaaccttgttctgtgattacggcttcgtggttatgcgaacgt
tcttacaaacgcaattgcacctgatgtaaaatcgtttttgctagctgtatggaacaagtgctcatgatgttcatgcaagatgcaattccagcttttgttggtttgtcatcttt
gtactgtgcttaccgcacataaagattgcatcttgcttattgctttgttgctttggtgctcgtccgcttctccttgcaccttatcaaacctttgtttagattctcttcttatagc
acttggtaactctcagctttacaacgccagtactgtttctgaaatttcatgactgataaagctgatagatggagtactaatatatgacatctttccataaatgttcgggtg
cagagatatggaggccccaggatcctatttacaggatgaacctacctgggccgctgtacgcatgacatccgcgagcaagtctgaggttctcaatgtacacatgaaatt
gatttttgctgcgtttggcttggctgatcgttgcatttgttctgattcatcagagttaaataacggatatatcagcaaatatccgcagcatccacaccgaccacacgtccg
gttaacagagtccccctgccttgctttaattattacggagtactccgctattaatccttagatatgtttcgaaggaactcaaaccttcctccatctgcaaatctcagtgctt
caaaactggaattagataattgaaaccttcattcggttgcaattcacaactgcaaattgaacagcactgtcaatttcaatttcgggttcacgattccaccgataggttga
catgatccatgatccacccattgtacaac

2B

ggcgtcaggactggcgaagtctggactctgcagggccgaactgctgaagacgaagcagaggaagagaaagggaagtgttcgacttgtaatttgtaggggtttttttta
gaggaacttgtaatttgtaggtgggctggcctcgttggaaaaacgatgctggctggttgggctgggccgatgtacgcttgcaaacaacttgtggcggcccgttctggac
gagcaggagtttctttttgttctcacttttctggtcttctttagttacggagtaccttttgtttttaaaggagttaccttttttttaggaattctttagttacctttcgcttgctc
tcaaaaaatatttaactttcgctttttttcattttaatttttgcaactatttacgagtttcatgaatgcttattttccagcatatcattatttgcaagtatttttatgccgtatgt
attggacgagagccatcgggactgttccagagactgcgtggtggggacggctcccaaccgccttttctatctctgttcgcatccggtggccgacttggctcgcgcgtga
gccgtgacgtaacagacttggtctcttccccatctggccatctataaattcccccatcgatcgaccctccctttccCCAATCCAGCACCCCCGATCCCGATCG
AAAATTCTCCGCAACAGCAAGCGATCGATCTAGCGAATCCCCGTCAAGgtatgtagcctctcgattcctcctcagccctgccctcgatttggtgta
cgcgttgagatgatgatctcgtagatgtctagatgacaccatgtcgatttgaaatagatcagatccgtgtagatcgatgagctcctgtgtacctgtggattcaagttattt
tcgcatgctattgttgtgatctactagatctagtgtgtgtattctatgctatcgatttctccgtgtagatttcactcgattactgttactgtggcttgatcggccatagatgtt
ggttaaggtttgatcggttagtgtttgaacctgcgtggatatctagcatccatctattatcgtgtaggtttcgaacaaacaagcactattattgtactgatggttcgtctat
ggttggttttgaccgttttagtgttgaacgagccttctgtatttgtttattgctgtccagtgatgtaccatgttcgttgagtgtcggattatactaattattgttgattgataat
cttgtagtttgcttttcctaatttatttatcgtagtcctgatttgcctcagctgtgcctcacccgtgcgatggtcaatcaacttgttagcccaatctgcttaatcatgtacattt
gttgttagaatcagagatcaagccaattagctatcttattgcttatctgttccatgttctgatcgatgtaacagtctacacttttgctctgtgctacttgattaaaacattct
gacttaaattcatgattggaagtttcagatctgattgttgccttacttgactaatatctattcatgtgacacctctctgtcttggtaacttaccgctgtttgtttgtaatttctg
actatgcag***ATG****cagatctttgtgaagaccctcactggcaaaaccatcacccttgaggtcgagtcgtccgacacgatcgacaacgtcaaggcaaagatccag*
gacaaggagggcattcctccagaccagcagcgcctcatctttgctggaaagcagcttgaggacggccgcaccctcgccgactacaacatccagaaggagtcc
accctccacctggtcctgaggctccgtggtggcatgcagatcttcgtcaagacccttaccggcaagaccatcacgctggaggtcgagtcctctgacacgatcgac
aatgtgaaggcgaagatccaggataaggagggcatcccccggaccagcagcgcctcatctttgccggcaagcagcttgaggacggccgtaccctcgccgac
tacaacatccagaaggaatccacactccatctggtgctcaggctgcgtggtggcatgcagatcttcgtcaagaccctaaccggcaagaccatcactctggaggt
tgagtcctctgacacgatcgacaatgtgaaggcaaagatccaggataaggagggcattcccccggaccagcagcgcctcatcttcgctggcaagcagcttga
ggatggccgcaccctggcagattacaatatccagaaggaatccaccttgcacctggtgcttcgcctccgtggtggcatgcagatctttgtaaagaccttgactgg
caagacaattaccctggaggttgagtcgtccgacacaattgacaatgtcaaggcgaagatccaggacaaggagggcatcccaccggaccagcagcgcctca
tcttcgccggcaagcagcttgaggatggtcgcacccttgcagattacaatatccagaaggaatccactctgcatctggtgcttcgtctccgcggtggaatgcaga
tcttcgttaagacgttgacagggaagaccatcacactggaggttgaatcttcggacaccattgacaacgtgaaggcaaagatccaggacaaggagggcatcc
ccccagaccagcagcgcctcatctttgctggtaagcagcttgaggatggccgcacccttgcagattacaacattcagaaggagtccaccctgcacctggtgctcc
*gtctccgtggtgggcag****TAA***gcttctgccgaactggttcacagtctgctgcccttggtggtctgccccttagtggtcatgccttttgttatgtgtcttgcgtcccaatcct
gtatcgtttgtgtgaacatctctgctgctgtatagcagcttgaatcctgttatgaatttgtgaacctgaaccttgttccgtgaatcatgttatgaataagtgaacctgaacc
ttgttccgtgattattgttacaatctgttgtgccgtatggttggtcgtgtgtgatttatgttgaactggagaaccaagttcgttccaggacatattgcaacctaagctaaac
catgtagaactacttgttctgggagacataaaacgtcatttttatgcattcgtaacatttaagcatactacaataattgtattgtccttttcctactcatccttgaaaccat
atgcctcttctcagcgcctctacatgcagtgtgctcagaacaaacaggccctgccagctgcttttcaattttccaattaataaccacaatagtcggactatggcatctgtg
ggtgactatgcaagatgttgctgtcaggtctctgaaacttttcccatgtatctgttgaaattacccagtaaattcatgcctctatttaatctggcatggttgattttcaaac
agaatgtgtttttttttgttctggaagctatattggtaaataaatacaaagctggagtgtgattatatttccaacagatattcaagaaaatctcagttgatttatttactac
tgtagtatatatatatatcttacagttgacttctcatatttcaaacgacatgtgagcacattgttcagtttcttaggatgtgttgtgtgctcaaaggtgtaattttgcattctg
ccctccgagtaaacactacacgtattttttgagtggcagtgcatttgattacaaggcaacaacaacaaaaacctatggcaagatatccttcttagaggctgccaggat
cattttgactgaactatgtaaggctgaagaaaagg

2C

tgcgtctggacgcacaagtcatagcattatcggctaaaatttcttaatttctaaattagtcatatcggctaagaaagtggggagcactatcatttcgtagaacaagaac
aaggtatcatatatatatatatatataatatttaaactttgttaagtggaatcaaagtgctagtattaatggagtttcatgtgcattaaattttatgtcacatcagcaattt
tgttgacttggcaaggtcatttagggtgtgtttggaagacaggggctattaggagtattaaacatagtctaattacaaaactaattgcacaaccgctaagctgaatcgc

gagatggatctattaagcttaattagtccatgatttgacaatgtggtgctacaataaccatttgctaatgatggattacttaggtttaatagattcgtctcgtgatttagcc
tatgggttctgctattaattttgtaattagctcatatttagttcttataattagtatccgaacatccaatgtgacatgctaaagtttaaccctggtatccaaatgaagtctta
tgagagtttcatcactccggtggtatatgtacttaggctccgttttcttccaccgacttatttttagcacccgtcacattgaatgtttagatactaattagaagtattaaacg
tagactatttacaaaatccattacataagacgaatctaaacggcgagacgaatctattaaacctaattagtccatgatttgacaatgtgttgctacagtaaacatttgct
aatgatggattaattaggcttaatagattcgtctcgccgtttagcctccacttatgtaatgggttttctaaacaatctacgtttaatactcctaattagtatctaaatattca
atgtgacacgtgctaaaaataagtcagtggaaggaagagaacgtccccttagttttccatcttattaattgtacgatgaaactgtgcagccagatgattgacaatcgca
atacttcaactagtgggccatgcacatcagcgacgtgtaacgtcgtgagttgctgttcccgtagAGAAATATCAACTGGTGGGCCACGCACATCAGCG
TCGTGTAACGTGGACGGAGGAGCCCCGTGACGGCGTCGACATCGAACGGCCACCAACCACGGAACCACCCGTCCCCACCTCTC
GGAAGCTCCGCTCCACGGCGTCGACATCTAACGGCTACCAGCAGGCGTACGGGTTGGAGTGGACTCCTTGCCTCTTTGCGCTG
GCGGCTTCCGGAAATTGCGTGGCGGAGACGAGGCGGGCTCGTCTCACACGGCACGGAAGACgtcacgggttccttccccacctctcctc
ttccccaccgccataaatagCCGACCCCCTCGCCTTTCTCCCCAATCTCATCTCGTCTCGTGTTGTTCGGAGCACACCACCCGCCCCAAA
TCGTTCTTCCCGCAAGCCTCGGCGATCCTTCACCCGCTTCAAGgtacggcgatcgtcttcctcctctagatcggcgtgatctgcaagtagttgatttg
gtagatggttaggatctgtgcactgaagaaatcatgttagatccgcgatgtttctgttcgtagatggctgggaggtggaatttttgtgtagatctgatatgttctcctgttt
atcttgtcacgctcctgcgatttgtggggattttaggtcgttgatctgggaatcgtggggttgcttctaggctgttcgtagatgaggtcgttctcacggtttactggatcatt
gcctagtagatcagctcgggctttcgtctttgtatatggtgcccatacttgcatctatgatctggttccgtggtgttacctaggtttctgcgcctgattcgtccgatcgatttt
gttagcatgtggtaaacgtttggtcatggtctgatttagattagagtcgaataggatgatctcgatctagctcttgggattaatatgcatgtgtcaccaatctgttccgtg
gttaagatgatgaatctatgcttagttaatgggtgtagatatatatgctgctgttcctcaatgatgccgtagcttttacctgagcagcatggatcctcctgttacttaggta
gatgcacatgcttatagatcaagatatgtactgctactgttggaattctttagtatacctgatgatcatccatgctcttgttacttgttttggtatacttggatgatggcatg
ctgctgctttttgttgatttgagcccatccatatctgcatatgtcacatgattaagatgattacgctgtttctgtatgatgccatagcttttatgtgagcaacatgcatcctc
ctggttatatgcattaatagatggaagatatctattgctacaatttgatgattattttgtacatacgatgatcaagcatgctcttcatactttgttgatatacttggataatg
aaatgctgctgcacgttcattctatagcactaatgatgtgatgaacacgcacgacctgtttgtggcatctgtttgaatgtgttgttgctgttcactagagactgttttatta
acctactgctagatacttacccttctgtctgtttattcttttgcag***ATG***cagatctttgtcaagaccctcaccggcaagaccatcaccctcgaggtggagtcttctgac
accattgacaacgtcaaggccaagatccaggacaaggaaggcattcccccggatcagcagcggctcatctttgccggcaagcagcttgaggatgggcgcacc
ctggctgactacaacatccagaaggagagcaccctccacctggtgctccgtctcaggggaggcatgcagatctttgtgaagaccttgactggcaagaccatca
cccttgaggtggagtcttccgacaccatcgacaacgtcaaggccaagatccaggacaaggagggcatcccccggaccagcagaggctcatctttgcgggca
agcagcttgaggatggacgcaccctggctgactataacatccagaaggagagcaccctccatctggtgctccgtctcaggggaggcatgcagatcttcgtgaa
gactctcactggcaagaccatcaccctcgaggtggagtcttccgacaccatcgacaacgtcaaggccaagatccaggacaaggagggcatcccccagacca
gcagaggctcatctttgctggcaagcagcttgaggacggacgcaccctggctgactataacatccagaaggagagcaccctccacctggtgctccgcctgagg
ggtgggatgcagatctttgtgaagactttgactggcaagaccattactttggaggttgagagctccgacaccatcgacaacgtgaaggccaagatccaggac
aaggaaggcatcccccggaccagcagaggctcatcttcgccggcaagcagcttgaggacggacgcaccctggctgactataacatccagaaggagagcac
cctccacctggtgctccgtctcaggggaggcatgcagatcttcgtgaagaccctcactggcaagaccatcacccttgaggtggagtcttccgacaccatcgacaa
tgtcaaggccaagatccaggacaaggagggcatcccccagaccagcagagactcatctttgcaggcaagcagcttgaggacggacgcaccctggctgact
acaacatccagaaggagagcaccctccacctggtgctccgtctcaggggaggcatgcagatcttcgtgaagaccctcactggcaagaccatcaccctcgaggt
ggagtcttctgacaccatcgacaacgtcaaggccaagatccaggacaaggagggcatcccccggaccagcagcgtcttatctttgccggcaagcagctgga
ggatggccgcacccttgcggattacaatatccagaaggagagcaccctccatctggtgctccgtctgaggggtgggatgcagatattcgtgaagactttgaccg
gcaagaccatcactttggaggttgagagctccgacaccattgacaatgtgaaggccaagatccaggacaaggagggtatcccccggaccagcagcgtctg
atcttcgccggcaagcaactggaggatggccgcaccctggcggactacaatatccagaaggagtccaccctccacctggtgctccgcctccgtggtggtcag***T
AA***gcccatcggtcatggatgcttctactgtacctgggtcgtctggtctctgcctgtgtcacctttgaagtacctgtgtcgggattgtgtttggtcatgaactgcagtttgtc
tttgatgttcttttgtctggtcttatgaactggttgtatctgtatgtttactgtaaactgttgttgcggtgcagcagtatggcatccgaatgaataaatgatgtttggactta
aatctgtactctgtttgttttcggttatgccagttctatattgcctgagatcagaatgtttagcttttgagttctgtttggcttgtggtcgactcctgtttcttacttgaggcgt
aactctgttctggcaaactcaaatgtctaactgaatgttttaggacttaattgttggacagattaacgtgtttggtttgtttctagattgtgattcggaaggcttgttagttg
tggaatcaaggagagcagctaggtctgtgcagaacgttattttggatttaagcccttctcagattatgccattactctaaacctaatgatatcatatttcactcggggatgt
tggagtagtcttttctttctcctgcagacaaaatgattttgctttcgtgtgtgtacatgattttgtgcaactgttgcaacaactgaagtagacaagttttgacctcaccaga
agaatgaaaaagattttggaatttgttacatcgacaaaccattgtaacttggcccatcagaatgcacagaagagcggctacaaattgacatgcgttgcaaactttgca
atagttgatgcacatgtttgccattgcctgccagtcttaggaaaagtgtgtggttcgagaaatctaagcatatgtgctctgctcacattgcgtggaacccacacagcttt

gtcacactcttgtccactccagaagtcattcctggcgctgtttacccctggtaaaaggtaaccgaaaacttctcaaggctgtacccaaaactggaaggaaatttggagg
aaatctttgcttttgatcggctcactctttc
