# Supplementary material for: Transcription Terminator-Mediated Enhancement in Transgene Expression in Maize: Preponderance of the AUGAAU Motif Overlapping With Poly(A) Signals
Source: Front Plant Sci. 2020 Oct 14;11:570778. doi: 10.3389/fpls.2020.570778 (PMC7591816; doi:10.3389/fpls.2020.570778)
Supplement: Supplementary Figure 3 — Nucleic acid alignment of the maize polyubiquitin1 (ZM Ubi1) gene promoter sequence with B. distachyon (Ubi1 and Ubi1-C) and S. italica (Ubi2) UBQ promoter sequences using Vector NTI software. The promoter sequences include the “total length” shown in the Supplementary Table 1. The highlighted nucleotides represent consensus among two (green), three (turquoise), or all four (yellow) sequences. All four sequence consensus is also highlighted with red font. [file DataSheet_3.pdf]

Supplementary Figure 3.

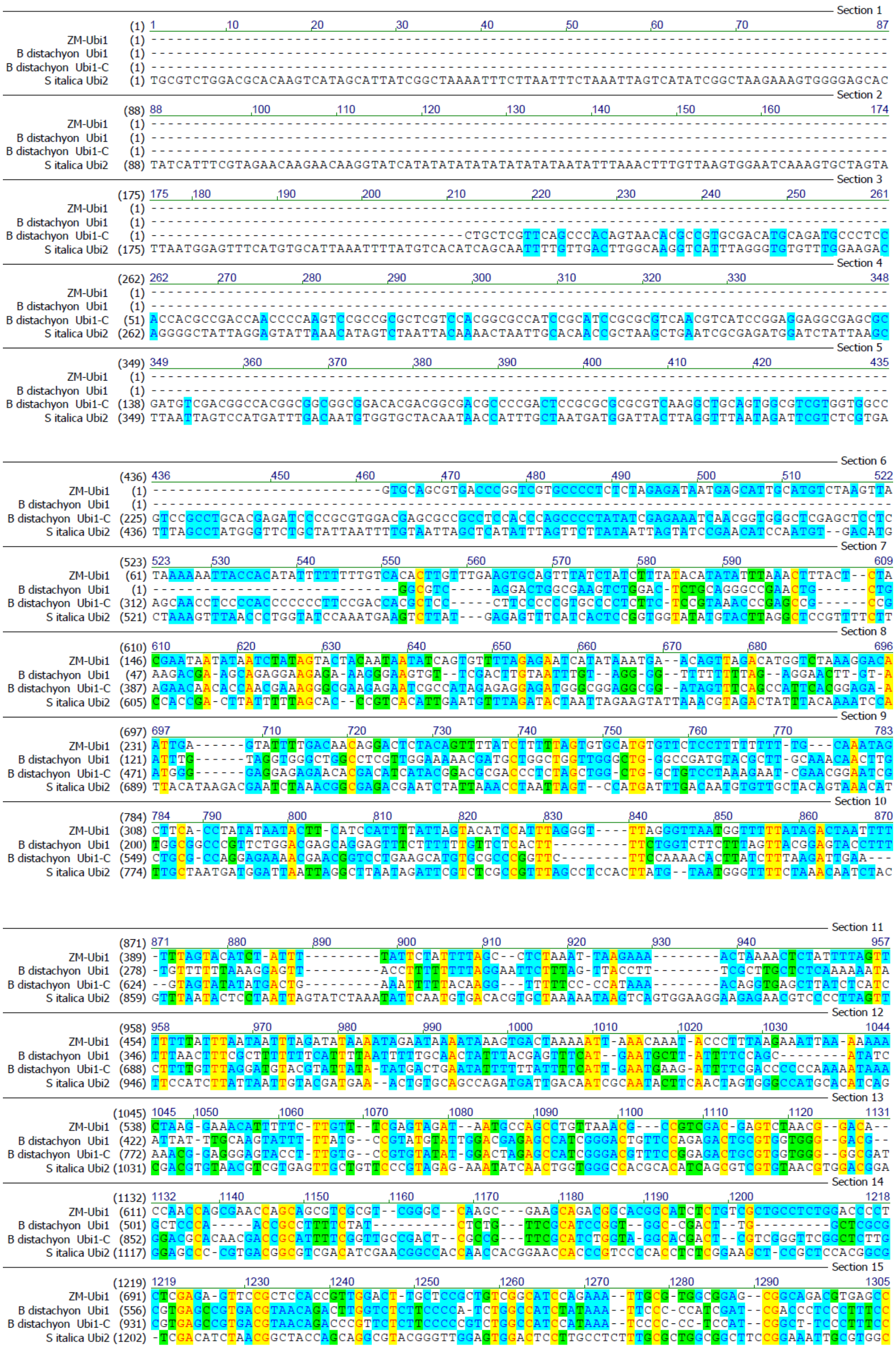

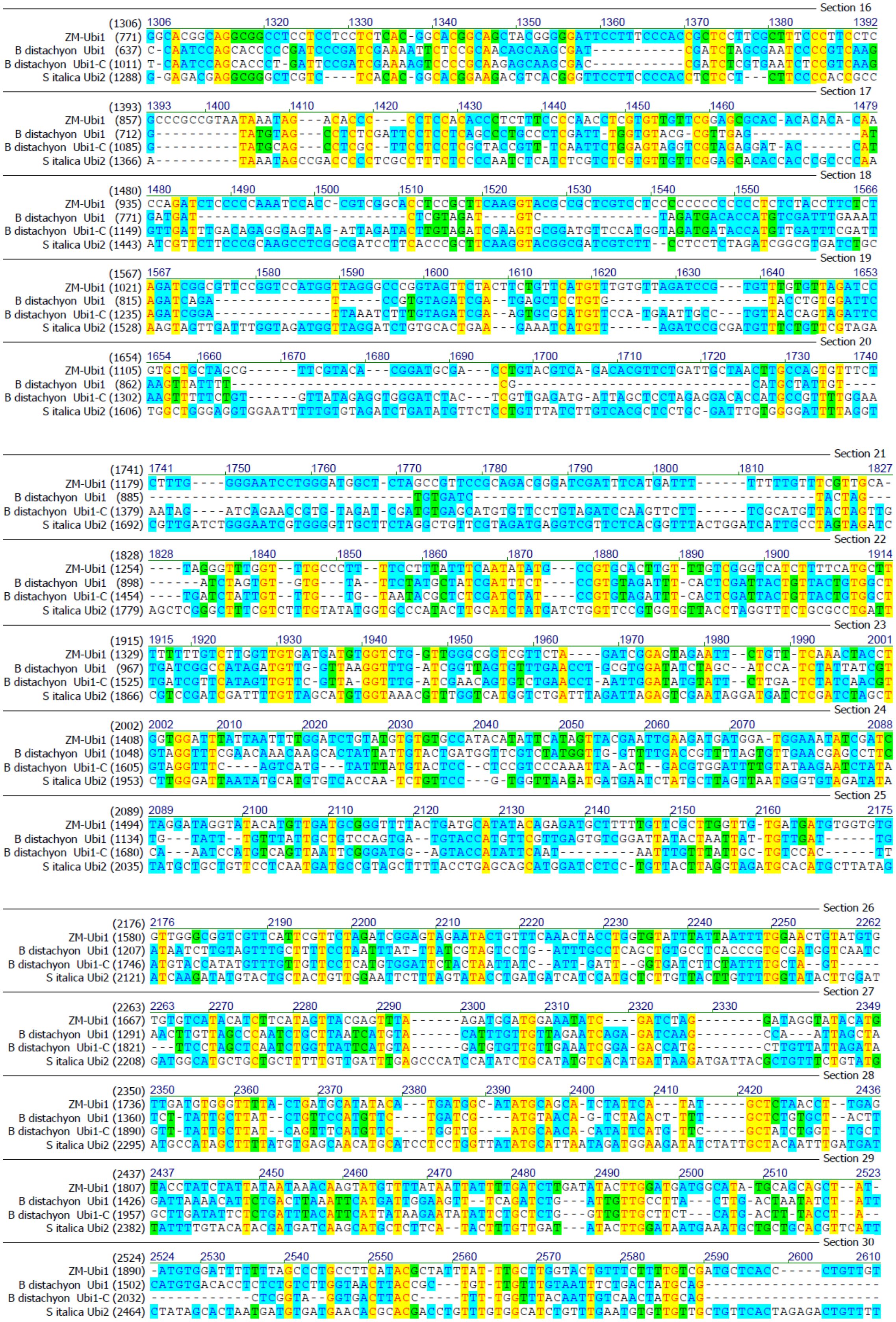

Section 16
Section 17
Section 18
Section 19
Section 20
Section 21
Section 22
Section 23
Section 24
Section 25
Section 26
Section 27
Section 28
Section 29
Section 30
ZM-Ubi1
B distachyon Ubi1
B distachyon Ubi1-C
S italica Ubi2

Section 31

(2611)	2611 2620 2630 2640 2650 2660
ZM-Ubi1 (1970)	TTGGTGTTACTTCTGCAG-------------------------------
B distachyon Ubi1 (1564)	---
B distachyon Ubi1-C (2078)	---
S italica Ubi2 (2551)	ATTAACCTACTGCTAGATACTTACCCTTCTGTCTGTTTATTCTTTTGCAG
