# Supplementary figures and images for: Transcription Terminator-Mediated Enhancement in Transgene Expression in Maize: Preponderance of the AUGAAU Motif Overlapping With Poly(A) Signals
Source: Front Plant Sci. 2020 Oct 14;11:570778. doi: 10.3389/fpls.2020.570778 (PMC7591816; doi:10.3389/fpls.2020.570778)

Supplementary Figure 4

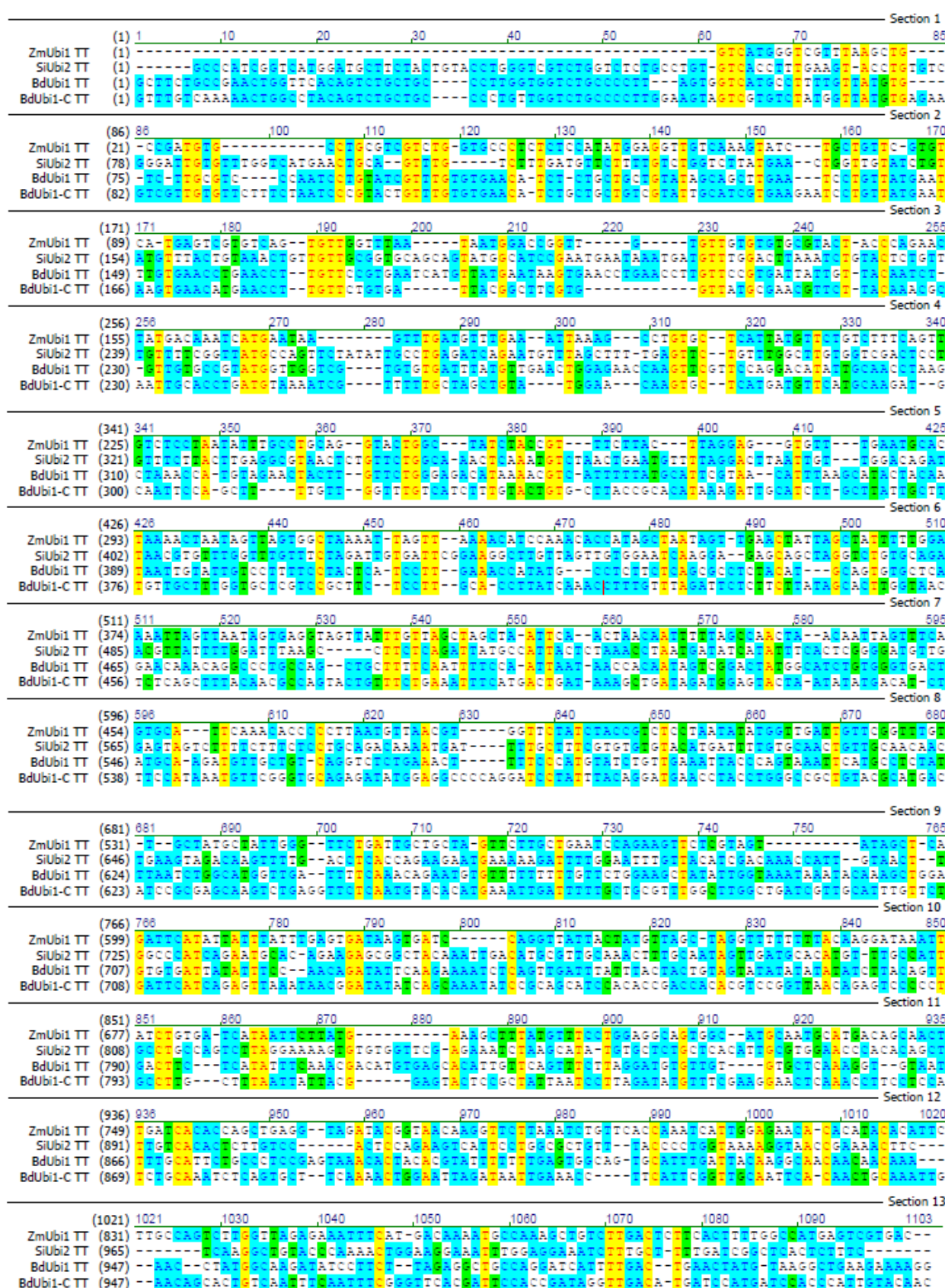

Supplement: Supplementary Figure 4 — Nucleic acid align of the maize polyubiquitin1 (ZmUbi1) gene TT sequence with putative TTs derived from the S. italica (SiUbi2) and B. distachyon (BdUbi1 and BdUbi1-C) UBQ genes. The alignment was carried out using Vector NTI software as described in Supplementary Figure 3. [file DataSheet_4.pdf]

Supplementary Figure 5

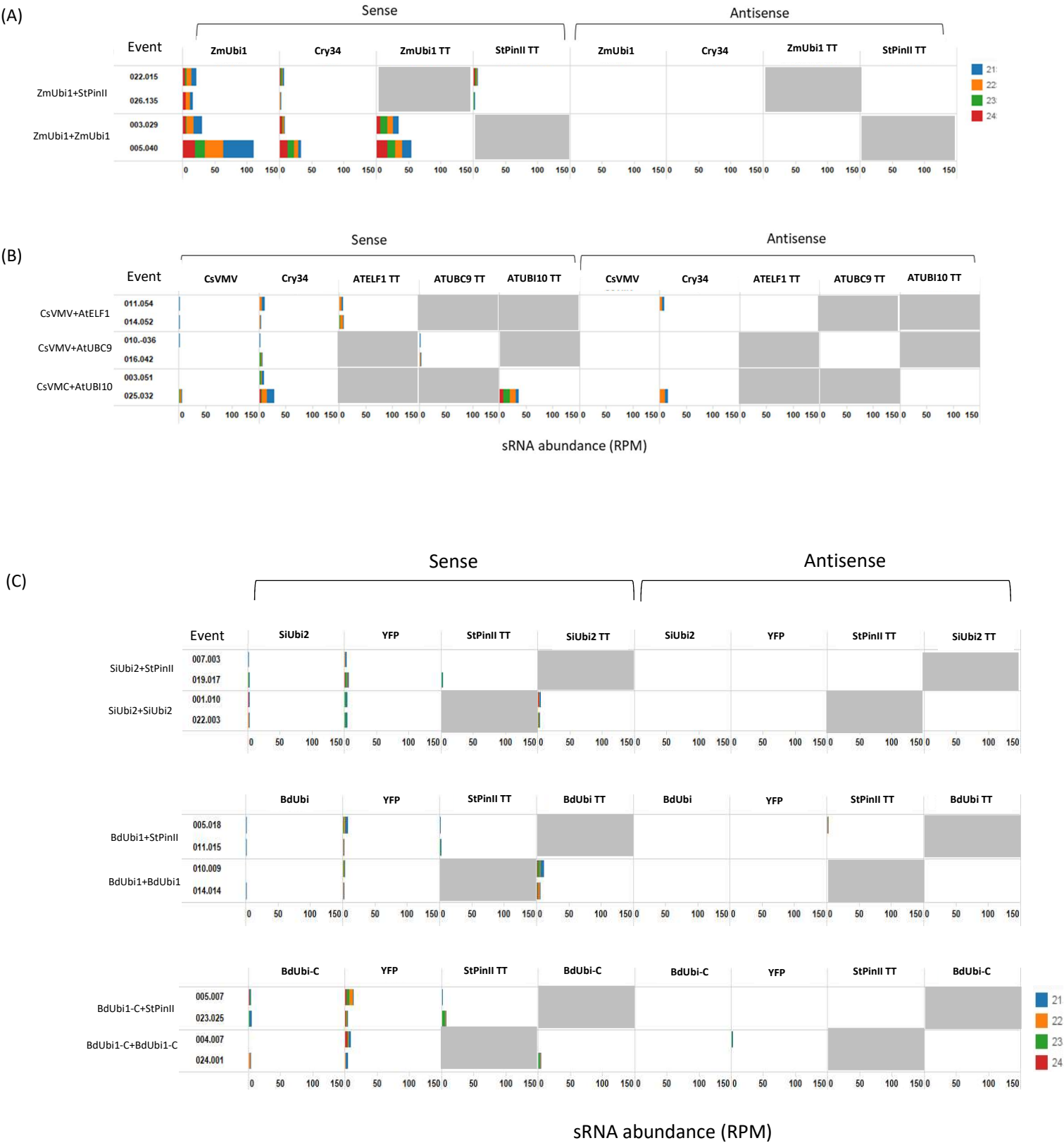

Supplement: Supplementary Figure 5 — siRNA analyses of distinct constructs. Sequencing of sRNA was performed as described in Materials and Methods, and the transgene-derived siRNA accumulation is shown here. For each construct, two independent events (as shown) were selected for whole-genome sRNA sequencing. The siRNA was mapped to each component of the transgene cassettes, including promoter (P), protein coding sequence, and TT with the differentiation of sense and antisense strand (denoted on top). The sRNA accumulation shown here is read counts normalized to the total reads derived from the corresponding library and depicted as stacked bars with colors signifying siRNA sizes (i.e., 21-24 nt). The comparison of siRNA accumulation is presented here for constructs ZmUbi1+StPinII (746) and ZmUbi1+ZmUbi1 (A); CsVMV+AtELF1, CsVMV+AtUBC9 and CsVMV+AtUbi10 (B); SiUbi1+StPinII, SiUbi1+SiUbi1, BdUbi1+StPinII, BdUbi1+BdUbi1, BdUbi1-C+StPinII and BdUbi1-C+BdUbi1-C (C). Grey highlighted areas represent the absence of particular RE in the construct of that event. [file DataSheet_5.pdf]
