# Supplementary material for: Transcription Terminator-Mediated Enhancement in Transgene Expression in Maize: Preponderance of the AUGAAU Motif Overlapping With Poly(A) Signals
Source: Front Plant Sci. 2020 Oct 14;11:570778. doi: 10.3389/fpls.2020.570778 (PMC7591816; doi:10.3389/fpls.2020.570778)
Supplement: Supplementary Table 1 — Length of the putative promoters and TTs for the UBQ genes. [file Table_1.pdf]

Supplementary Table 1

	Putative promoter				
**Promoter Name**	**TSS[A] and upstream elements (bp)**	**5' UTR (bp)**	**Intron (bp)**	**Total promoter Length (bp)[C]**	**Total Transcription Terminator length (bp)**
ZmUbi1	895	82	1010	1987	910
BdUbi1	636	75	874	1585	1020
BdUbi1-C	1029	55	993	2077	1026
SiUbi2	1064	373[B]	1114	2599	1039

[A]TSS: transcription start site. [B]5' UTR sequence was interrupted by another 48 bp intron, which is included in the total length. [C]Sum of preceding three columns.
