# Supplementary material for: Transcription Terminator-Mediated Enhancement in Transgene Expression in Maize: Preponderance of the AUGAAU Motif Overlapping With Poly(A) Signals
Source: Front Plant Sci. 2020 Oct 14;11:570778. doi: 10.3389/fpls.2020.570778 (PMC7591816; doi:10.3389/fpls.2020.570778)
Supplement: Supplementary Table 2 — Putative FUE motifs identified in various TTs that have been reported previously to function as 3′ end processing signals. A subset of the motifs observed in the rice and Arabidopsis gene FUEs and putative stabilization signals are also depicted. The underlined sequences show nt variations in previously reported sequences. [file Table_2.pdf]

Supplementary Table 2

Construct	Transcription Terminator	Putative FUE motif[A]	Subset of the FUE motifs	Putative mRNA Stabilization signal[A]	References
117, 118, 119, 746	StPinII	AAUGUAUG (63)<br>UGUGG (103)<br>UGUGUG (128)<br>UGUAAU (139)	AAUGUA[1,2]<br>UGUAUG[1,2]<br>UGUGG[2,3]<br>GUGUG[1,2,3]<br>UGUGUG[1,2]<br>UGUAAU[1,2]	CGUGUC UU[4] (207)	[1]Shen et al., 2008; [2]Loke et al., 2005; [3]Wu et al., 1993; [4]An et al., 1989
401	SiUbi2	CAUGG (38)<br>UGUGUUU (110)<br>UUGUA (171)<br>UGUAUG (178)<br>UGUAAA (189)	CAUGG[3]<br>UGUGUUU[2,5]<br>UUGUA[1,2,5]<br>UGUAUG[1,2]<br>UGUAAA[1,2]		[1]Shen et al., 2008; [2]Loke et al., 2005; [3] Wu et al., 1993; [5] Hunt 1994
402	BdUbi1	GGUGG (63)<br>UGUGUGAA (127)<br>AUUUGUGAA (174)	GGUGG[1,2,5]<br>UGUGU[1,2,3]<br>GUGUG[1,2,3]<br>UGUGAA[1,2]<br>UGUGUG[1,2]<br>GUGUGA[1,2,3]<br>AUUUGU[1,2]		[1]Shen et al., 2008; [2]Loke et al., 2005; [3]Wu et al., 1993; [5] Hunt 1994
400	BdUbi1-C	UGUGUGAA (140)	UGUGU[1,2,3]<br>GUGUG[1,2,3]<br>UGUGAA[1,2]<br>UGUGUG[1,2]<br>GUGUGA[1,2,3]	CGUGUC UA[4] (87)	[1]Shen et al., 2008; [2]Loke et al., 2005; [3] Wu et al., 1993; [4]An et al., 1989
744	ZmUbi1	CAUGG (30)<br>CAGUGUUGGUUUA AUAAUG (128)<br>GUGUUGUGUGUG (155)	CAUGG[1,2,3]<br>CAGUGUUGG UUU[3]<br>UAAUAAUG[3]<br>GUGUUG[1,2,3]<br>UGUUGUG[2,5]<br>GUGUG[1,2,3]<br>UGUGUGUG[1,2]	CGUGUC A[4] (111)<br><br>CGUGUC AG[4] (123)	[1]Shen et al., 2008; [2]Loke et al., 2005; [3]Wu et al., 1993; [4]An et al., 1989; [5]Hunt 1994
806	AtELF1	CAGUGUUAGUUU (64)<br>GGUGG (171)<br>UUUUUUGCUUU (201)<br>AUUUGU (214)	CAGUGUUAG UUU[3]<br>GGUGG[1,2,5]<br>UUUUUU[1,2]<br>UUUGCUUU[2.5]<br>AUUUGU[1,2]	CGUGUC UG[4] (102)	[1]Shen et al., 2008; [2]Loke et al., 2005; [3]Wu et al., 1993; [4]An et al., 1989; [5]Hunt 1994;
807	AtUBC9	UAAUAAUU (49)<br>UAUGUAAU (74)<br>AUUUGUGAA (112)<br>UUGUA (163)	UAAUAAUU[3]<br>UAUGUA[1,2]<br>UGUAAU[1,2]<br>AUUUGU[1,2]		[1]Shen et al., 2008; [2]Loke et al., 2005; [3]Wu
